# Neurogranin Promotes Neuronal Maturation and Network Activity Through Ca^2+^/Calmodulin Signaling

**DOI:** 10.3390/ijms27073306

**Published:** 2026-04-06

**Authors:** Elena Martínez-Blanco, Raquel de Andrés, Esperanza López-Merino, José A. Esteban, Francisco Javier Díez-Guerra

**Affiliations:** 1 Group of Molecular Basis of Neuronal Plasticity, Departamento de Biología Molecular, Instituto de Biología Molecular (IUBM), Centro de Biología Molecular, Consejo Superior de Investigaciones Científicas, Universidad Autónoma de Madrid, 28049 Madrid, Spain; 2 Group of Mechanisms of Synaptic Plasticity, and Contribution to Cognitive Function, Centro de Biología Molecular (CSIC-UAM), 28049 Madrid, Spain; esperanza.lopez@cib.csic.es (E.L.-M.); jaesteban@cbm.csic.es (J.A.E.)

**Keywords:** Neurogranin, calcium, calmodulin, neuronal maturation

## Abstract

Neurogranin (Ng) is a postsynaptic calmodulin-binding protein highly enriched in forebrain neurons and widely implicated in synaptic plasticity. However, whether Ng contributes more broadly to neuronal network maturation and cellular homeostasis remains unclear. Here, we examined the consequences of silencing or restoring Ng to adult physiological levels in primary hippocampal neurons. Ng expression promoted dendritic expansion, increased synaptic number, and shifted the axon initial segment toward the soma, consistent with structural adaptations to enhanced connectivity. Calcium (Ca^2+^) imaging revealed a marked increase in spontaneous neuronal activity and network synchronization, which was confirmed by electrophysiological recordings showing enhanced burst firing and spike synchrony. At the molecular level, Ng altered Ca^2+^/calmodulin (CaM) signaling by increasing total CaM levels, reducing Ca^2+^/CaM-dependent protein kinase II (CaMKII) abundance while increasing its relative autophosphorylation, and downscaling specific ionotropic glutamate receptors. Despite elevated network activity, Ng expression enhanced neuronal metabolic competence and viability, reduced cellular stress signaling and induced modest caspase-3 activation without engagement of apoptotic pathways. Together, these results indicate that Ng promotes neuronal maturation and coordinated network activity while engaging compensatory mechanisms that preserve excitatory balance and neuronal resilience. Our findings identify Ng as a molecular integrator linking Ca^2+^/CaM signaling with the structural and functional maturation of neuronal networks.

## 1. Introduction

Neurogranin (Ng) is a small postsynaptic protein highly enriched in principal neurons of the forebrain [1,2], where it is predominantly localized to the somatodendritic compartment and dendritic spines [3]. Ng binds calmodulin (CaM) through a central IQ motif [4], an interaction that is favored at low intracellular calcium (Ca^2+^) concentrations. Phosphorylation of Ng by protein kinase C (PKC) [5] disrupts this interaction, thereby regulating the availability of free CaM for downstream signaling pathways. Through this mechanism, Ng has long been proposed to act as a modulator of Ca^2+^/CaM signaling in neurons, influencing the activation of CaM-dependent enzymes involved in synaptic plasticity [6,7]. Among neuronal CaM-binding proteins, Ng is particularly abundant in the forebrain [8] and has been proposed to regulate the spatiotemporal dynamics of Ca^2+^/CaM signaling within dendritic spines [9]. By binding apo-CaM under resting Ca^2+^ conditions and releasing it upon Ca^2+^ elevation, Ng may shape the availability of CaM for downstream signaling cascades. Computational and experimental studies [10,11,12] have suggested that this mechanism can influence the activation threshold of CaM-dependent targets such as Ca^2+^/CaM-dependent protein kinase II (CaMKII), thereby modulating synaptic plasticity and activity-dependent signaling. Through this capacity to buffer and redistribute CaM, Ng is thought to influence Ca^2+^-dependent synaptic signaling [13]. Consistent with this role, numerous studies have linked Ng to mechanisms of learning and memory. Mice lacking Ng display alterations in hippocampal synaptic plasticity and spatial learning [14,15], and Ng levels in humans correlate with cognitive performance [16]. In addition, Ng has emerged as a sensitive biomarker of synaptic dysfunction in neurological disorders, particularly Alzheimer’s disease, where its levels are reduced in brain tissue but elevated in cerebrospinal fluid (CSF) [17,18,19,20,21]. Together, these observations highlight Ng as an important component of neuronal signaling pathways that support synaptic function and cognitive processes.

Despite extensive investigation of its biochemical properties and role in synaptic plasticity, it remains unclear whether Ng functions solely as a local regulator of Ca^2+^/CaM signaling at individual synapses or whether it also contributes more broadly to the coordinated structural and functional maturation of neuronal networks. In particular, little is known about how Ng expression influences neuronal connectivity, spontaneous network activity, and the compensatory adaptations that maintain excitatory balance during neuronal maturation. Studies using Ng knockout mice have reported differing effects on hippocampal plasticity, including impaired Long-Term Potentiation (LTP) in some paradigms [14] and enhanced LTP with reduced Long-Term Depression (LTD) in others [22]. These discrepancies highlight the complexity of Ca^2+^/CaM-dependent signaling and suggest that Ng function may depend strongly on cellular context, activity patterns, or developmental stage.

Neuronal development involves the progressive emergence of coordinated network activity, driven by the interplay between synaptic connectivity, intrinsic excitability, and activity-dependent signaling pathways [23,24]. Ca^2+^-dependent signaling plays a central role in this process, regulating dendritic growth, synapse formation, and the stabilization of functional circuits [25,26]. Proteins that modulate Ca^2+^/CaM signaling may therefore influence not only individual synapses but also the maturation of neuronal networks. Notably, Ng expression increases relatively late during brain development, coinciding with periods of intense synaptogenesis and circuit remodeling [1,2,27]. This temporal pattern raises the possibility that Ng contributes to the maturation and stabilization of developing neuronal networks rather than to earlier stages of neuronal differentiation.

Primary cultures of hippocampal neurons provide a useful system to address these questions, as they allow controlled manipulation of gene expression and detailed analysis of neuronal morphology, activity, and survival. Interestingly, we previously observed that Ng expression in mature hippocampal neuron cultures remains substantially lower than in adult brain tissue [28]. This observation provides an opportunity to experimentally restore Ng expression to physiological levels and examine its consequences for neuronal development and network function. Using adeno-associated viral vectors (AAVs) to drive Ng expression selectively in excitatory neurons, we investigated how Ng affects dendritic growth, synaptic connectivity, spontaneous neuronal activity, and cellular resilience in hippocampal cultures. Our results indicate that Ng promotes coordinated structural and functional maturation of neuronal networks while engaging compensatory mechanisms that maintain excitatory balance and neuronal viability.

## 2. Results

### 2.1. Neurogranin Promotes Structural Maturation and Synaptic Connectivity

**Figure 1:**
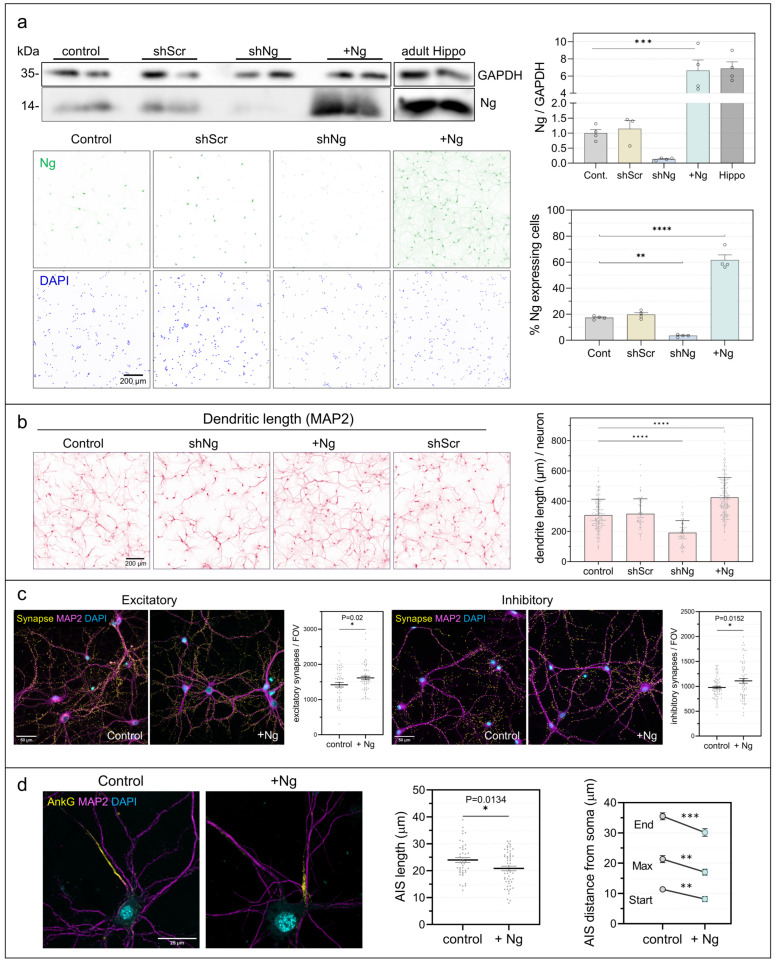
Ng expression modulates neuronal morphology and synapse density. Primary hippocampal neurons were infected with AAV-Ng at DIV7 or with AAV-shNg or scrambled shRNA (AAV-shScr) at DIV10 and analyzed at DIV15. (**a**) Ng protein levels were assessed by Western blot and normalized to GAPDH (upper panel; n = 4). The proportion of Ng-positive neurons was determined by immunofluorescence using DAPI to identify total cells (lower panel; n = 4). (**b**) Dendritic length per neuron, quantified for each field of view, increased following Ng expression. (**c**) Synaptic density was analyzed by co-localization of excitatory (vGluT1/PSD95) and inhibitory (GAD65/Gephyrin) pre- and postsynaptic markers. Data in histograms are total number of synapses per field of view (0.11 mm^2^). Ng expression increased the number of excitatory and inhibitory synaptic contacts (yellow puncta; n = 52–58 images). (**d**) The axon initial segment (AIS) was labeled with anti-ankyrin G (AnkG). Total AIS length (left graph; n = 25 neurons per condition) and distances from soma to AIS onset, peak intensity, and distal end (right graph) were quantified using DAPI to locate the soma. Widefield images (**a**–**c**) were acquired on a Zeiss Axiovert 200M microscope with 10× (**a**,**b**) or 40× (**c**) objectives; AIS images (**d**) were obtained using a Zeiss LSM900 confocal microscope with a 63× objective. Statistical analysis was performed using an unpaired Student’s *t*-test. * *p* < 0.05, ** *p* < 0.01, *** *p* < 0.001, **** *p* < 0.0001.

To investigate how Ng expression influences neuronal maturation and network function, we established experimental conditions allowing controlled manipulation of Ng levels in cultured hippocampal neurons. As shown in Figure 1a, baseline Ng expression in DIV15 hippocampal cultures was approximately fivefold lower than in adult hippocampus, consistent with our previous observations [28]. This reduced endogenous expression provides a useful baseline for assessing the consequences of restoring Ng to physiologically relevant levels. Infection with AAV-Ng restored Ng abundance to adult levels and markedly increased the proportion of Ng-expressing neurons, whereas shRNA-mediated knockdown reduced Ng protein to barely detectable levels. In the AAV-Ng construct, Ng expression is driven by the CaMKIIα promoter, ensuring selective expression in excitatory neurons. These manipulations were confirmed both biochemically and by quantification of Ng-positive neurons, establishing a robust framework for analyzing the consequences of Ng expression during neuronal development.

Morphological analysis revealed pronounced structural changes following both Ng expression and knockdown (Figure 1b). AAV-mediated Ng expression significantly enhanced dendritic growth, whereas shNg markedly reduced it. Quantification of total dendritic length per neuron confirmed these effects: dendritic length was substantially decreased by shNg, significantly increased by AAV-Ng, and unaffected by scrambled shRNA. We next examined synaptic number in control and AAV-Ng–infected cultures by quantifying the colocalization of pre- and post-synaptic markers (Figure 1c). Ng expression increased the number of both excitatory and inhibitory synapses, indicating that Ng promotes synaptogenesis and synapse stabilization during neuronal maturation in vitro. Finally, we analyzed the axon initial segment (AIS) in control and AAV-Ng–infected neurons. As shown in Figure 1d, Ng expression slightly shortened the AIS and shifted it closer to the soma. Such AIS remodeling may represent an adaptive response to increased synaptic connectivity. Together, these results indicate that restoring Ng to physiological levels in excitatory neurons promotes coordinated structural remodeling, including enhanced dendritic growth, increased synapse number, and repositioning of the AIS, whereas Ng depletion produces the opposite effects. These findings suggest that Ng contributes to the structural maturation of neuronal networks and provide a foundation for subsequent functional analyses.

### 2.2. Neurogranin Promotes Spontaneous Neuronal Activity and Network Synchronization

**Figure 2:**
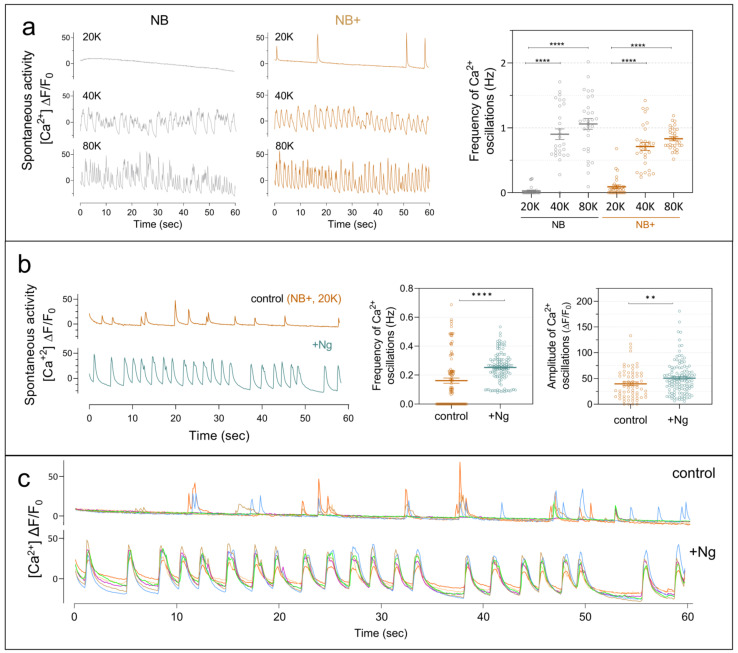
Ng expression enhances spontaneous calcium oscillations in hippocampal neurons. (**a**) Representative spontaneous Ca^2+^ oscillations detected using the jGCaMP8s biosensor in DIV15 hippocampal neurons cultured in NB or NB+ medium at different plating densities. Higher neuronal density increased the frequency of Ca^2+^ oscillations in both media. (**b**) Representative traces of spontaneous calcium activity in NB+ medium show increased frequency and amplitude of oscillations in Ng-expressing neurons (n = 100 neurons). (**c**) Synchronous Ca^2+^ activity across all neurons in the same field was observed following Ng expression. Spontaneous calcium activity of five individual neurons is represented in different colors. Statistical analysis was performed using two-way ANOVA. ** *p* < 0.01, **** *p* < 0.0001.

Having observed that Ng expression promotes dendritic growth and increases synaptic connectivity, we next examined whether these structural changes translate into altered neuronal activity. Hippocampal neurons in culture begin firing action potentials from approximately DIV7 onwards [29,30], but robust spontaneous activity typically emerges only at high plating densities. Because the low-density conditions required for detailed morphological analysis limit spontaneous network activity, we first assessed the influence of plating density on neuronal activity using the genetically encoded calcium indicator jGCaMP8 (Figure 2a). At the density routinely used for our structural studies (20,000 cells/cm^2^), neurons displayed no detectable activity in Neurobasal™ (NB) medium and only sparse, low-frequency events in Neurobasal™ Plus (NB+) medium, which modestly enhances neuronal activity. In contrast, cultures plated at higher densities (40,000 and 80,000 cells/cm^2^) exhibited robust spontaneous activity, consistent with previous observations [30]. These results confirm that low-density cultures provide a suitable baseline for detecting activity changes resulting from Ng expression.

Under these conditions, neurons expressing Ng exhibited markedly increased activity compared with controls, with higher frequency and amplitude of calcium transients (Figure 2b). In addition, Ng expression promoted strong synchronization of activity across neurons (Figure 2c). These findings indicate that Ng expression is sufficient to enhance spontaneous neuronal activity and promote coordinated network activity even in sparsely connected cultures.

**Figure 3:**
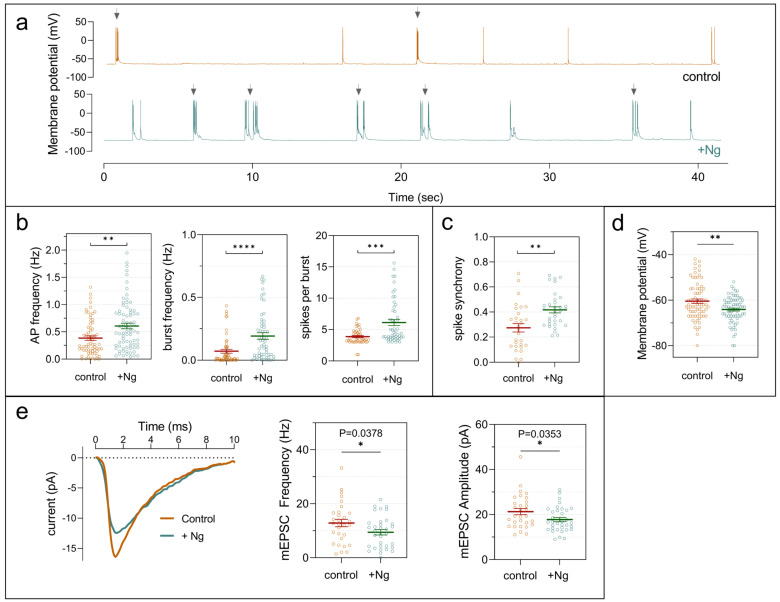
Ng expression increases spontaneous electrical activity and network synchronization. Hippocampal neurons cultured in NB+ medium were analyzed at DIV18 by whole-cell patch-clamp. (**a**) Representative 40-s current-clamp traces showing spontaneous action potential (AP) bursts (arrows) in control and Ng-expressing neurons. (**b**) Quantification of AP frequency, burst frequency, and APs per burst (n = 70–75 neurons). (**c**) Spike synchrony was assessed by simultaneous recordings from neuron pairs as described in Section 4. (**d**) Resting membrane potential was more hyperpolarized in Ng-expressing neurons. (**e**) Miniature excitatory postsynaptic currents (mEPSCs) were recorded in the presence of 1 µM TTX and 10 µM bicuculline. Representative averaged mEPSCs are shown (left), and histograms depict frequency and amplitude distributions (right). Statistical analysis was performed using an unpaired Student’s *t*-test. * *p* < 0.05, ** *p* < 0.01, *** *p* < 0.001, **** *p* < 0.0001.

To confirm these observations with higher temporal resolution, we performed whole-cell patch-clamp recordings. At DIV18, Ng-expressing neurons fired action potentials at a higher rate than control neurons (Figure 3a). Spiking activity frequently occurred in bursts or short high-frequency trains, with both burst incidence and the number of action potentials per burst significantly increased in Ng-expressing neurons (Figure 3b). Paired recordings further revealed enhanced spike synchrony, as measured using a ±2.5 ms coincidence window (Figure 3c). Despite this elevated firing and synchronization, Ng expression was associated with intrinsic membrane properties indicative of reduced excitability. Ng-expressing neurons exhibited a more hyperpolarized resting membrane potential (Figure 3d), and miniature excitatory postsynaptic currents (mEPSCs) exhibited lower frequency and amplitude compared with controls (Figure 3e). Together, these results demonstrate that Ng expression promotes increased firing, burst activity, and network synchronization despite reduced intrinsic excitability. These findings suggest that Ng-driven structural maturation enhances synaptic connectivity and enables efficient coordination of neuronal activity even in low-density cultures.

### 2.3. Neurogranin Reshapes Ca^2+^/CaM-Dependent Signaling Pathways

**Figure 4:**
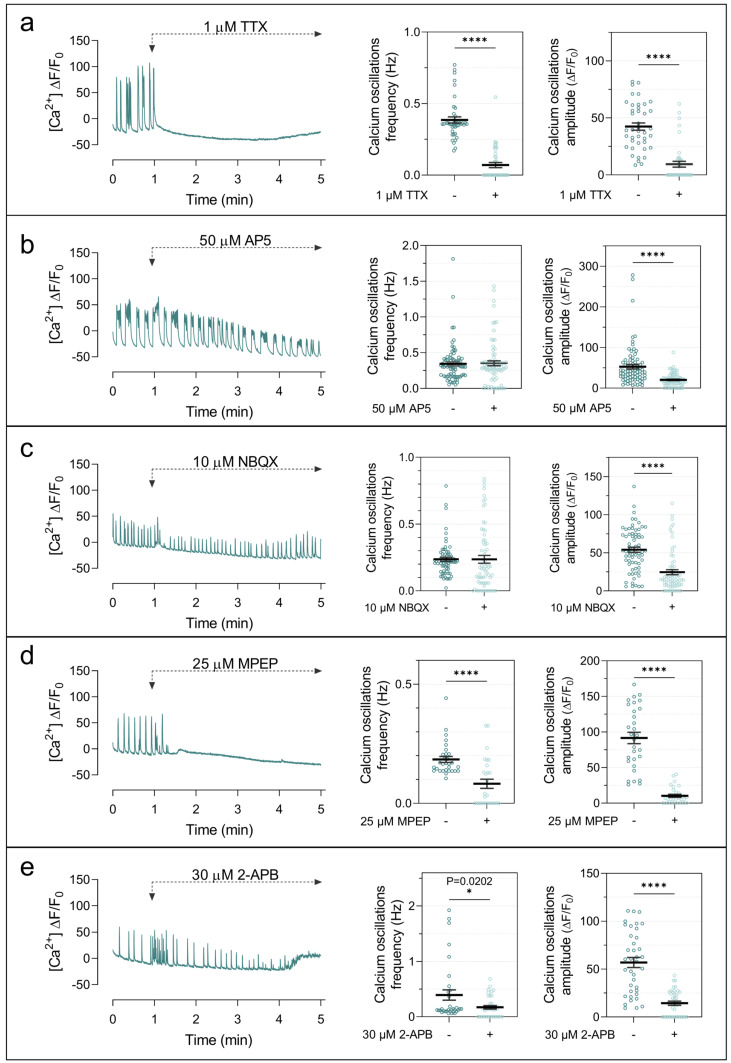
Synaptic signaling mediates Ng-induced increases in spontaneous calcium activity. Spontaneous DIV15 hippocampal neurons infected with AAV-Ng and maintained in NB+ medium from DIV7 were monitored for spontaneous Ca^2+^ oscillations. Baseline activity was recorded for 1 min, followed by acute application of pharmacological agents: 1 µM TTX (**a**), 50 µM AP5 (**b**), 10 µM NBQX (**c**), 25 µM MPEP (**d**), or 30 µM 2-APB (**e**). Ca^2+^ activity was then recorded for 2 min, starting 1 min after treatment. Representative calcium traces are shown, and histograms compare oscillation frequency and amplitude before and after drug application. Between 30 and 90 cells were analyzed per condition. Statistical analysis was performed using an unpaired Student’s *t*-test. * *p* < 0.05, **** *p* < 0.0001.

To elucidate the mechanisms underlying the Ng-dependent increase in network activity, we first examined the signaling pathways contributing to the Ca^2+^ transients observed in Ng-expressing neurons. Calcium imaging experiments combined with pharmacological manipulations revealed that the enhanced activity depends on synaptic transmission and intracellular calcium mobilization. Application of tetrodotoxin (TTX), a voltage-gated sodium channel blocker, completely abolished Ca^2+^ transients in Ng-expressing neurons (Figure 4a), demonstrating that the activity requires action potential firing. We next assessed the contribution of glutamatergic receptors. Short-term NMDA receptor blockade with AP5 selectively reduced the amplitude of high-calcium events without affecting firing frequency (Figure 4b), whereas inhibition of AMPA receptors with NBQX reduced both event amplitude and firing frequency (Figure 4c). These results indicate that ionotropic glutamatergic signaling contributes to both the magnitude and frequency of network activity.

In addition, inhibition of mGluR5 with MPEP markedly reduced both the frequency and amplitude of Ca^2+^ transients (Figure 4d). Because mGluR5 activates phospholipase C signaling, leading to IP_3_-mediated Ca^2+^ release from intracellular stores, we tested the IP_3_ receptor inhibitor 2-APB. This treatment strongly reduced Ca^2+^ event frequency and amplitude (Figure 4e), indicating that intracellular Ca^2+^ release contributes to the enhanced activity observed in Ng-expressing cultures. Together, these findings indicate that Ng-dependent network activity involves coordinated contributions from ionotropic glutamatergic transmission and metabotropic receptor-dependent intracellular calcium signaling.

**Figure 5:**
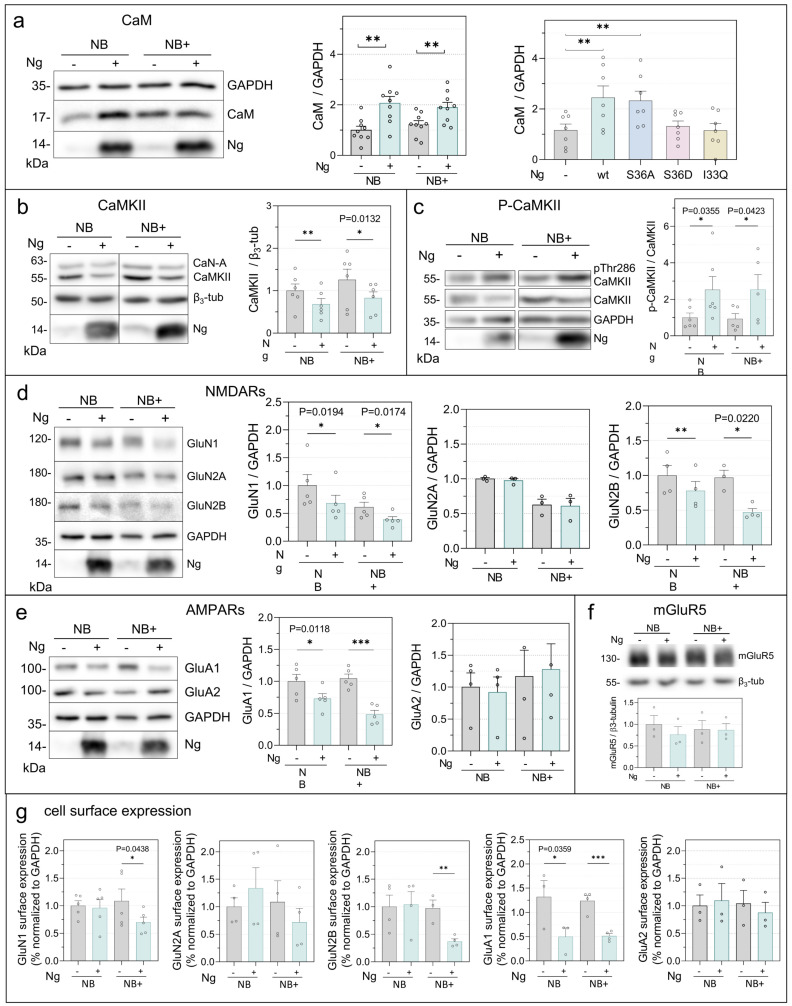
Ng expression alters synaptic protein abundance and surface expression. Primary hippocampal neurons were infected with AAV-Ng and maintained in NB or NB+ medium from DIV7. Protein extracts at DIV16 were analyzed by Western blot, and levels were normalized to GAPDH or β3-tubulin. Histograms show mean normalized values relative to non-infected neurons in NB. (**a**–**c**) CaM, total CaMKII, and phospho-CaMKII (Thr286) were analyzed. (**d**,**e**) NMDA receptor subunits (GluN1, GluN2A, GluN2B) and AMPA receptor subunits (GluA1, GluA2) were assessed. (**f**) mGluR5 levels were analyzed. (**g**) Surface expression of NMDA and AMPA receptors was determined by sulfo-NHS-biotin labeling followed by streptavidin–agarose isolation. GAPDH served as a control for the input fraction (n = 4). Statistical analysis was performed using paired and unpaired Student’s *t*-test. * *p* < 0.05, ** *p* < 0.01, *** *p* < 0.001.

We next investigated how Ng expression influences Ca^2+^/CaM-dependent signaling pathways. Ng binds CaM and has been proposed to regulate CaM availability in neurons [7,31]. In both NB and NB+ media, Ng expression significantly increased total CaM levels (Figure 5a), with a stronger effect in NB medium, which exhibits lower baseline activity and reduced neuronal survival. Analysis of Ng mutants revealed that only CaM-binding-competent variants (Ng-wt and Ng-Ser36Ala) increased CaM levels, whereas CaM-binding-deficient mutants (Ser36Glu and Ile33Gln) had no effect, supporting a role for Ng–CaM interaction in regulating CaM abundance.

We then examined Ca^2+^/CaM-dependent protein kinase II (CaMKII), a central mediator of synaptic plasticity [32]. Ng expression reduced total CaMKII levels but significantly increased the ratio of phosphorylated Thr286-CaMKII to total CaMKII (pCaMKII/CaMKII) (Figure 5b,c), indicating a shift toward a higher relative activation state. This effect was observed only with CaM-binding-competent Ng variants (Figure S1). In contrast, levels of the Ca^2+^/CaM-dependent phosphatase calcineurin (CaN-A) remained unchanged (Figure 5b and Figure S2). These results indicate that Ng expression alters the balance of CaM-dependent signaling pathways.

Finally, we examined whether these signaling changes were accompanied by alterations in synaptic receptor composition. Ng expression selectively reduced both total and surface levels of the NMDA receptor subunits GluN1 and GluN2B, whereas GluN2A levels were unchanged (Figure 5d,g). Similarly, total and surface levels of the AMPA receptor subunit GluA1 were reduced, whereas GluA2 and mGluR5 expression remained unchanged (Figure 5e–g). Surface expression closely paralleled total protein levels, indicating that Ng selectively downscales specific excitatory receptor subunits.

Together, these results indicate that Ng expression promotes a coordinated reorganization of Ca^2+^/CaM-dependent signaling and synaptic composition. By modulating CaM availability, Ng enhances CaMKII activation while simultaneously reducing the abundance of specific ionotropic glutamate receptors. These adjustments likely represent compensatory adaptations that stabilize network activity while limiting postsynaptic excitability.

### 2.4. Neurogranin Promotes Neuronal Metabolic Competence and Survival

**Figure 6:**
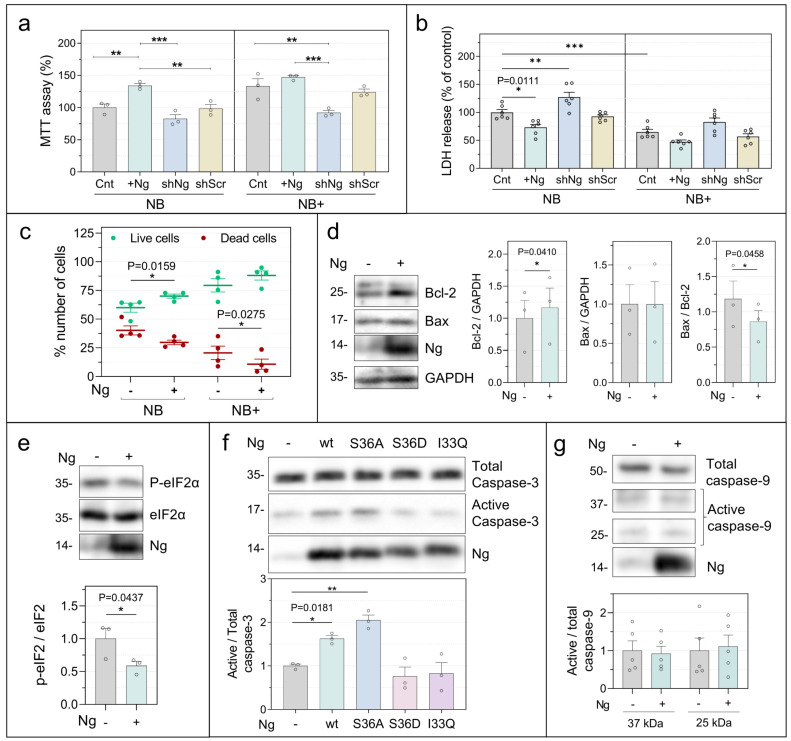
Ng expression promotes neuronal viability and stress resilience. Primary hippocampal neurons were infected with AAV-Ng or AAV-shNg and maintained in NB or NB+ medium from DIV7. (**a**) Mitochondrial metabolic activity was assessed at DIV16 using the MTT assay. (**b**) Cytotoxicity and membrane integrity were assessed using the lactate dehydrogenase (LDH) release assay. (**c**) Neuronal viability was evaluated using a combined Calcein-AM/Propidium Iodide (PI)/Hoechst 33342 assay at DIV16. Live cells were identified by Calcein-AM fluorescence, dead cells by PI staining, and total cell number by Hoechst 33342 labeling (1 µg/mL). (**d**) Expression of the anti-apoptotic protein Bcl-2 and the pro-apoptotic protein Bax was analyzed in neuronal extracts at DIV16 and normalized to GAPDH. (**e**) Phosphorylation of eIF2α at Ser51, an indicator of integrated stress response activation, was analyzed by Western blot and normalized to total eIF2α levels. (**f**,**g**) Levels of active (cleaved) caspase-3 and caspase-9, respectively, were quantified and normalized to their respective total protein levels. Data are presented as mean values ± SEM relative to control conditions (n = 4 independent experiments). Statistical analysis was performed using paired and unpaired Student’s *t*-test. * *p* < 0.05, ** *p* < 0.01, *** *p* < 0.001.

Because Ng expression markedly increased neuronal activity and network synchronization, we next examined whether these changes influence neuronal viability. Metabolic activity was first assessed using the MTT assay (Figure 6a). In NB medium, Ng expression significantly increased mitochondrial reducing activity, whereas Ng knockdown produced only a modest, non-significant reduction. In NB+ medium, which supports higher baseline metabolic activity, the most pronounced effect was observed following Ng knockdown, which significantly decreased metabolic activity.

We next evaluated membrane integrity using the lactate dehydrogenase (LDH) release assay (Figure 6b). Ng expression consistently reduced LDH release, whereas Ng knockdown increased LDH levels in both NB and NB+ media, indicating enhanced membrane damage and cytotoxicity. Similar results were obtained using the calcein-AM/propidium iodide (PI) assay, which simultaneously measures intracellular esterase activity in live cells and membrane permeability in dead cells (Figure 6c). Quantification of live and dead cells revealed that Ng expression increased the proportion of viable neurons and reduced cell death under both culture conditions. This protective effect was particularly evident at DIV23, when neuronal viability typically declines in culture (Figure S3).

To investigate the mechanisms underlying this enhanced viability, we analyzed markers of cellular stress and regulators of the intrinsic apoptotic pathway. Ng expression did not alter levels of the pro-apoptotic protein Bax but produced a modest yet significant increase in the anti-apoptotic protein Bcl-2, resulting in a reduced Bax/Bcl-2 ratio (Figure 6d). In parallel, Ng expression significantly decreased the phosphorylation of eIF2α at Ser51, a key mediator of the integrated stress response (Figure 6e), consistent with reduced cellular stress signaling.

We also examined caspase-3 activation. Neurons expressing CaM-binding Ng variants (Ng-wt and Ng-Ser36Ala) exhibited a modest increase in active caspase-3 levels (Figure 6f). Because Ng expression increased neuronal viability, this activation is unlikely to reflect apoptotic execution. Instead, moderate caspase-3 activation has been associated with non-apoptotic roles in synaptic plasticity and structural remodeling [33,34,35,36,37,38]. Consistent with this interpretation, Ng expression did not alter levels of active caspase-9, an initiator of the intrinsic apoptotic pathway (Figure 6g). Notably, the caspase-3 effect was restricted to CaM-binding Ng variants, which also increased CaM levels and elevated the pCaMKII/CaMKII ratio. Together, these results indicate that Ng expression enhances neuronal metabolic competence and resistance to stress while engaging controlled remodeling pathways rather than apoptotic signaling. Importantly, the levels of Ng achieved by AAV-mediated expression closely match those found in adult hippocampal neurons in vivo, underscoring the physiological relevance of these effects.

## 3. Discussion

In this study, we identify Ng as a key regulator of neuronal maturation that links Ca^2+^/CaM signaling with the coordinated structural and functional development of neuronal networks. By combining morphological analysis, calcium imaging, electrophysiology, biochemical measurements, and multiple viability assays, we show that restoring Ng expression to physiological levels promotes dendritic growth and synaptic connectivity, enhances spontaneous neuronal activity and network synchronization, and engages adaptive signaling programs that support neuronal metabolic competence and survival. Importantly, these effects depend on Ng’s ability to bind CaM, establishing a mechanistic connection between Ca^2+^-dependent signaling and the structural and functional maturation of neuronal circuits.

Using cultured hippocampal neurons as a model system, we examined the impact of Ng expression at three levels of neuronal organization: structural maturation, network activity, and neuronal viability. AAV-mediated Ng expression restored protein levels comparable to those observed in the adult hippocampal tissue and was selectively restricted to excitatory neurons through the CaMKIIα promoter. This experimental design allowed the effects of Ng to be evaluated within a physiologically relevant expression range during a defined developmental window characterized by active synaptogenesis. Our results indicate that Ng promotes structural maturation of hippocampal neurons. Ng expression increased dendritic arbor complexity, enhanced synaptic number, and repositioned the axon initial segment closer to the soma. Dendritic architecture is a major determinant of neuronal information processing because neurons dynamically adjust their morphology in response to activity and synaptic input [39]. In cultured hippocampal neurons, synaptogenesis and dendritic growth increase markedly during the second and third weeks in vitro [40], and both processes depend strongly on neuronal activity and Ca^2+^-dependent signaling pathways [25,26]. Under the relatively low-density culture conditions used here, where spontaneous connectivity and activity are limited, Ng expression markedly enhanced dendritic development and synaptic connectivity. These observations suggest that Ng facilitates developmental maturation programs that promote the emergence of integrated neuronal networks.

### 3.1. CaM Signaling and Compensatory Synaptic Regulation

A substantial body of experimental and computational work indicates that Ng binds free CaM (apo-CaM) within dendritic spines and modulates local Ca^2+^/CaM signaling during synaptic activity [10,11,13,41]. By concentrating CaM at synapses, Ng is proposed to amplify activity-dependent Ca^2+^ signals, such that each synaptic Ca^2+^ influx generates higher local Ca^2+^/CaM levels and facilitates activation of downstream effectors with relatively low Ca^2+^/CaM affinity, most notably CaMKII [42]. Sustained engagement of this mechanism during neuronal maturation would be expected to promote synaptic potentiation, synaptogenesis, and stabilization of dendritic arbors. Consistent with this framework, Ng expression increased the relative activation state of CaMKII, as reflected by elevated Thr286 phosphorylation.

However, our results also reveal that Ng does not simply enhance excitatory signaling. In addition to increasing CaMKII activation, Ng expression reduced total CaMKII abundance, downscaled specific ionotropic glutamate receptor subunits, hyperpolarized the resting membrane potential, and reduced mEPSC amplitude. Together, these observations indicate a coordinated reorganization of CaM-dependent signaling and synaptic composition. Such adjustments resemble mechanisms associated with homeostatic plasticity [43], in which sustained increases in network activity elicit compensatory changes that stabilize neuronal output while preventing excessive excitation. The elevated CaMKII phosphorylation observed here further aligns with previous findings in Ng knockout mice, in which basal CaMKII autophosphorylation is markedly reduced [14]. These complementary gain- and loss-of-function observations support a model in which Ng regulates the availability of Ca^2+^/CaM to tune the basal activation state of CaMKII and thereby shape synaptic signaling dynamics.

Together, our data support the view that Ng acts primarily through its interaction with CaM to influence multiple aspects of neuronal maturation. However, the precise downstream pathways linking Ng-dependent Ca^2+^/CaM regulation to dendritic growth, synaptic remodeling, and network synchronization remain to be defined. Given the large number of CaM-regulated targets, further studies will be needed to identify the specific molecular effectors involved.

### 3.2. Cooperative Ionotropic and Metabotropic Signaling

Pharmacological analysis of Ca^2+^ transients further revealed that Ng-dependent network activity relies on coordinated contributions from ionotropic and metabotropic glutamate receptors. Inhibition of AMPA or NMDA receptors significantly reduced Ca^2+^ signals, indicating that spontaneous activity arises from synaptically driven glutamatergic transmission. Unexpectedly, inhibition of mGluR5 produced an even stronger suppression of Ca^2+^ activity, markedly reducing both event frequency and amplitude. Because mGluR5 activates Gq/11-coupled phospholipase C signaling and IP_3_-mediated Ca^2+^ release from intracellular stores, these findings indicate that intracellular Ca^2+^ mobilization contributes substantially to the enhanced activity observed in Ng-expressing cultures.

Notably, Ng expression did not alter total mGluR5 protein levels, suggesting that enhanced receptor engagement or downstream signaling efficiency accounts for this effect. The increased burst firing observed in Ng-expressing neurons may facilitate activation of perisynaptic mGluR5 receptors, which require sustained glutamate release for effective signaling. Together, these observations support a model in which Ng amplifies spontaneous network activity through cooperative ionotropic–metabotropic signaling and intracellular Ca^2+^ mobilization, rather than simply by increasing excitatory synaptic drive.

### 3.3. Neuronal Resilience and Adaptive Remodeling

Despite markedly altering neuronal firing patterns, Ng expression did not compromise neuronal viability. Instead, Ng promoted metabolic competence and cellular resilience, as evidenced by increased mitochondrial activity, reduced membrane damage, enhanced cell viability, and decreased phosphorylation of the stress marker eIF2α. These findings indicate that the increased network activity induced by Ng is accompanied by adaptive cellular responses that support neuronal stability.

Interestingly, Ng expression also induced a modest increase in active caspase-3 without activating the upstream initiator caspase-9, indicating that the intrinsic apoptotic pathway is not engaged. Moderate caspase-3 activation is increasingly recognized as a component of non-apoptotic neuronal remodeling, contributing to synaptic plasticity, dendritic restructuring, and circuit refinement [34,37,44,45,46,47]. In this context, the controlled caspase-3 activation observed in Ng-expressing neurons may contribute to structural remodeling processes that accompany increased network activity, including synaptic pruning or dendritic branch refinement. The restriction of this effect to CaM-binding Ng variants further suggests that it may arise from activity-dependent signaling downstream of Ca^2+^/CaM pathways.

Collectively, these findings indicate that Ng couples increased neuronal activity and connectivity to adaptive programs that maintain cellular homeostasis. In this framework, moderate caspase-3 activity may serve a sculpting role that refines synaptic architecture while preserving neuronal viability.

### 3.4. Implications for Neuronal Development and Disease

Previous work from our group demonstrated that Ng promotes synapse formation in cultured neurons [28], and Han et al. [48] showed that Ng coordinates experience-dependent synapse elimination and the conversion of silent synapses into functional AMPAR-containing synapses during developmental critical periods. Consistent with these roles, Ng expression increases relatively late during brain development [2], coinciding with periods of intense synaptogenesis and circuit remodeling rather than earlier stages of neuronal proliferation or migration.

Alterations in Ng expression have also been linked to cognitive performance and neurological disease. Reduced Ng levels have been reported in several conditions associated with cognitive impairment, including hypothyroidism [49,50], schizophrenia [51], and Alzheimer’s disease [52]. Conversely, elevated Ng levels in cerebrospinal fluid, reflecting synaptic loss, correlate with disease progression and cognitive decline in Alzheimer’s disease [18,20]. Together, these observations highlight Ng as an important regulator of synaptic development and circuit refinement. In summary, this study identifies Ng as a molecular integrator that links Ca^2+^/CaM signaling to the coordinated structural, functional, and homeostatic maturation of neuronal networks.

## 4. Materials and Methods

### 4.1. Animals and Ethics Compliance

Wistar rats were bred at the animal facility of the Universidad Autónoma de Madrid (UAM). All procedures conducted during the study strictly adhered to the Spanish Royal Decree 1201/2005, which governs the protection of animals used in scientific research, as well as the European Union Directive 2010/63/EU [53] concerning the welfare of animals in scientific contexts. Experimental protocols were approved by the corresponding institutional and regional ethics committees, ensuring that the highest standards of animal care and ethical compliance were maintained throughout the research. The animal experimentation procedures used in the present study were approved by the “Animal Experimentation Ethics Committee of the Centro de Biología Molecular Severo Ochoa” (CEEA-CBMSO-23/247, date: 10 October 2019), the “Ethics Committee for Animal Experimentation of the Autonomous University of Madrid” (CEI 98-1828-A302, date 19 November 2019) and the “Animal Protection Area, Directorate-General for Agriculture, Livestock and Food, Regional Ministry for the Environment and Spatial Planning” (PROEX 106/19, date 9 May 2019).

### 4.2. Reagents

Fetal bovine serum (FBS), Dulbecco’s modified Eagle’s medium (DMEM), 0.25% trypsin, Neurobasal media (NB) and B27 supplement were from Thermo Fisher Scientific Inc. (Waltham, MA, USA). The protease inhibitor cocktail was from Biotools (B14001) (Madrid, Spain). Total protein was measured using the Bradford Protein Assay kit (Bio-Rad, Alcobendas, Madrid, Spain). Pre-stained protein markers VI (10-245 kDa) were from PanReac-AppliChem (Castellar del Vallès, Barcelona, Spain). Immobilon-P membranes and ECL Western blotting reagents were from Millipore (Burlington, MA, USA). 1-beta-arabino-furanosylcytosine (AraC) was from Calbiochem (251010) (San Diego, CA, USA). AP5 was from Tocris (ref. 0106) (Bristol, UK), 2-APB and Poly-L-lysine hydrobromide (PLL) from Sigma-Aldrich (St. Louis, MO, USA) (refs. 100065 and P2636, respectively), NBQX was from Tocris (ref. 0373), TTX from Alomone Labs (Jerusalem, Israel) (ref. T-500) and MPEP from MedChemExpress (Monmouth Junction, NJ, USA) (ref. HY-14609A). Paraformaldehyde (PFA) was from Merck (Darmstadt, Germany). Antibodies and plasmids used in this study are listed in Tables S1–S3.

### 4.3. Primary Cultures of Rat Hippocampal Neurons

Primary hippocampal neurons were prepared from embryonic day 19 (E19) Wistar rat embryos as previously described [54]. Embryos were collected and maintained in chilled Hank’s balanced salt solution (HBSS) and hippocampi were carefully dissected free of meninges. Tissue was washed five times in HBSS and incubated with 0.25% trypsin for 15 min at 37 °C to facilitate cell dissociation. After trypsin removal by two additional washes in HBSS, tissue was transferred to HBSS containing 1.26 mM CaCl_2_, 0.81 mM MgSO_4_ and 0.04 mg/mL DNase I and dissociated mechanically using Pasteur pipettes and 22 G needles. The resulting cell suspension was filtered through a 70 µm nylon mesh, centrifuged at 300× *g* for 5 min, resuspended in plating medium (10% FBS/DMEM, 1 mM Pyruvate, 10 mM D-glucose), and counted. Cells were plated at a density of 25,000 cells/cm^2^ onto culture dishes pre-coated with 0.1 mg/mL PLL in borate buffer (pH 8.0), or at 12,000 or 20,000 cells/cm^2^ on 18-mm or 25-mm coverslips, respectively, pre-treated with 0.25 mg/mL PLL. Coverslips were previously sterilized with 65% nitric acid for 24–72 h, extensively washed with double-distilled H_2_O (ddH_2_O) and baked at 180 °C. Then, 3 h after plating to allow cell adhesion to the substrate, the plating medium was replaced with Neurobasal medium (NB) supplemented with B27 and GlutaMAX (Thermofisher). To suppress glial proliferation, 1 µM AraC was added on day in vitro 3 (DIV3). On DIV7, 50% of the culture medium was replaced with fresh NB/B27. Cultures were maintained at 37 °C in a humidified atmosphere with 5% CO_2_ and used for experiments during the third week in vitro. All treatments were performed within the thermostatized CO_2_ incubator.

### 4.4. Preparation of Lentiviral and Adeno-Associated Viral Particles

HEK-293T cells were cultured in DMEM supplemented with 10% FBS (Gibco) and maintained at 37 °C with 5% CO_2_. Passaging was performed twice a week.

Lentiviral particles (LVs) production: All lentiviral constructs are derived from the vector pLOX-Syn-DsRed-Syn-GFP [55], kindly donated by Dr. FG Scholl. HEK-293T cells (3 × 10^6^) were seeded in p100 dishes and transfected 24 h later with 8 µg of the lentiviral plasmid of interest, 4 µg of pCMVδR8.74, and 2 µg of pMD2.G using PEI MAX (DNA:PEI ratio 1:2). DNA and PEI were mixed in OptiMEM medium (Gibco), incubated for 20 min at room temperature (RT), and added dropwise to the cells. After 5 h of incubation at 37 °C and 5% CO_2_, the transfection medium was replaced with Neurobasal medium. After 48 h, the medium containing lentiviral particles was collected, filtered through a 0.45 µm filter, aliquoted, and stored at −80 °C. Cultures of hippocampal neurons were infected at day in vitro 4 (DIV4).

Adeno-associated virus (AAV) production: AAVs were prepared as previously described [56]. HEK-293T cells (7 × 10^6^) were seeded in p150 dishes and cultured in 10% FBS/DMEM until 70% confluency, at which point the medium was replaced with 5% FBS/DMEM. Cells were then transfected using the calcium phosphate method with the following plasmids: 12.5 µg of the desired pAAV plasmid, 25 µg of pFδ6, 6.25 µg of pH21, and 6.25 µg of pRV1. Plasmids were diluted and adjusted to a volume of 900 µL using CaCl_2_ (final concentration 125 µM). An equal volume of 2× HBS (274 mM NaCl, 10 mM KCl, 1.4 mM Na_2_HPO_4_, 15 mM D-Glucose and 42 mM HEPES buffer, pH 7.05) was added, and the solution mix bubbled and was incubated for 20 min at RT and then added dropwise to the cultures. After 16 h of incubation (37 °C, 5% CO_2_), the medium was replaced by 10% FBS/DMEM. Then, 48 h post-transfection, cells were washed with PBS and lysed in extraction buffer (150 mM NaCl, 20 mM Tris-HCl pH 8, 0.4% sodium deoxycholate, 50 U/mL benzonuclease (Millipore)). Lysates were centrifuged at 3000× *g* for 15 min, and the supernatant applied to a heparin Sepharose column (HiTrap, Cytiva). Eluted fractions were concentrated using Amicon Ultra-4 100 kDa filters (Millipore), filtered through 0.13 µm filters, aliquoted, and stored at −80 °C. Hippocampal neurons were typically infected at DIV7 with AAV particles diluted in half the culture volume of the dish. After an 8-h incubation, the viral medium was replaced with a 1:1 mixture of the previously collected medium and fresh NB supplemented with B27 and GlutaMAX. To silence Ng expression, the plasmid shNg pA_RC3J1_CAGW (Addgene #92155), encoding an shRNA targeting the sequence gtgacaagacttccctactgt, was used. Prior to shNg AAV production, the GFP coding region of the original plasmid was replaced by mRuby2. As a control, a non-targeting scrambled shRNA (gtgccaagacgggtagtca, Addgene #181875) was used. To preserve neuronal viability, AAV infections for knockdown experiments were performed at DIV10.

### 4.5. Protein Extraction and Western Blots

Cells were lysed in extraction buffer containing 50 mM NaCl, 0.5% Triton X-100, 1 mM EDTA, 2 mM DTT, 25 mM Tris-HCl (pH 6.8), and protease & phosphatase inhibitors (Biotool). Lysates were homogenized by 20 passes through a 23G needle and centrifuged at 17,500× *g* for 15 min at 4 °C. Protein concentrations in the supernatants were determined using the Bradford assay (Bio-Rad) and 15–25 μg from hippocampal neuron extracts were loaded per well. Proteins were separated by SDS-PAGE on 6%, 10% or 13% polyacrylamide gels (Mini Vertical Protein Electrophoresis System, Cleaver Scientific) under reducing conditions at 120 V for ~2.5 h. Protein in the gels was transferred onto PVDF membranes (Millipore) using a semi-dry transfer system (Nyx Technik) at a constant current of 400 mA for 30 min in transfer buffer (22.5 mM Tris, 170 mM glycine, 20% methanol). Membranes were blocked with 5% (*w*/*v*) skimmed milk in TBS for 1 h at room temperature with agitation and incubated overnight at 8 °C with primary antibodies in TBS with 0.05% Tween-20. HRP-conjugated secondary antibodies (Jackson ImmunoResearch, 1:15,000) were used for detection with an enhanced chemiluminescence system (ECL, Millipore). Signal acquisition was performed using an Amersham Imager 680 (GE Healthcare Life Sciences), and densitometric analysis was carried out with the open source software Fiji/ImageJ version 2.16/1.54s [57,58].

### 4.6. Cell Surface Biotinylation

Hippocampal neurons at DIV16, previously seeded in 6-well plates, were washed with ice-cold PBS and cell surface proteins were labeled for 30 min at 4 °C by incubation in a 1 mL solution containing the non-permeable Sulfo-NHS-SS-Biotin (1 mg/mL in PBS). The biotinylation reagent was subsequently quenched twice with 100 mM L-Lysine for 30 min at 4 °C. After three additional washes with PBS, cells were lysed for 30 min in lysis buffer (150 mM NaCl, 1 mM EDTA, 50 mM Tris-HCl (pH 7.4), 1% Triton X-100 and proteases inhibitors), and the lysate was clarified by centrifugation at 17,500× *g* for 15 min. Biotinylated proteins were recovered by incubating the cleared lysate with streptavidin agarose beads for 90 min at room temperature. After washing the beads three times with 1 mL of the lysis buffer, bound proteins were eluted in 2× Laemmli sample buffer at 95 °C, separated by SDS-PAGE, and analyzed in Western blot.

### 4.7. Immunofluorescence

Primary hippocampal neurons (DIV16–17) grown on 18 mm round coverslips were quickly washed with PBS and fixed with 4% PFA in PBS for 20 min at room temperature. The fixative was removed, and the cells incubated with 0.2 M glycine (pH 8) for 5 min at RT to quench residual PFA. After three PBS washes, cells were permeabilized and blocked for 30 min in blocking buffer containing 0.1% Triton X-100, 1% bovine serum albumin (BSA), and 1% heat-inactivated horse serum in PBS. Incubation with primary antibodies (Table S3) was conducted overnight at 8 °C in PBS supplemented with 1% BSA and 1% horse serum. After three 5-min PBS washes, secondary antibodies were applied for 1 h at room temperature in the same buffer. Nuclei were counterstained with DAPI (0.2 μg/mL in PBS) for 5 min, followed by washes in distilled water and 96% ethanol. Coverslips were air-dried and mounted using Mowiol. Images were acquired on a Zeiss AxioObserver7 fluorescence microscope and a Hamamatsu Orca Fusion Camera. Excitatory and inhibitory synaptic contacts were identified by immunostaining with antibodies against specific presynaptic and postsynaptic markers (Figure 1c). MAP2 labeling was used to define dendritic compartments and to restrict synapse analysis to regions in close apposition to dendrites. Synaptic contacts were analyzed and quantified using the SynapTrack [59] macro language script implemented in Fiji/ImageJ [57,58].

### 4.8. Measurement of Axon Initial Segment (AIS) Length and Distance from the Soma

Hippocampal neurons were processed for immunofluorescence using antibodies against Ankyrin G (AnkG) and MAP2 and imaged with a Zeiss LSM900 laser-scanning confocal microscope equipped with a 63× PlanApo NA 1.4 objective. Image stacks consisted of 6–8 optical sections acquired at 0.37 μm z-intervals. Z-stacks were projected into single images using maximum intensity projection in Fiji/ImageJ. The AIS was identified by AnkG immunoreactivity, and the neuronal soma was defined based on DAPI staining. A line region of interest (ROI) was then drawn from the soma along the AnkG-positive axon to delineate the AIS, following the method described by Grubb & Burrone (2010) [60]. Fluorescence intensity profiles along the ROI were smoothed over 1 μm intervals using SigmaPlot 12.5 and normalized to the maximal AnkG fluorescence, which was set to 100%. The proximal and distal boundaries of the AIS were defined as the points at which the normalized AnkG signal declined to 33% of the maximum at each end. AIS length was calculated as the distance between these two points, and the distance from the soma was defined as the distance between the soma and the proximal boundary.

### 4.9. Calcium Imaging

Hippocampal neurons were plated on poly-L-lysine–coated 25 mm round coverslips at a density of 20,000 cells/cm^2^ and infected at DIV4 with lentiviral particles encoding mRuby2-jGCaMP8s, enabling expression of the calcium indicator jGCaMP8s [61] and the calcium-insensitive fluorescent protein mRuby2 [62], both under the control of CaMKIIα promoters. AAVs were added at DIV7. At DIV15, cultures were washed and incubated for 15 min at 37 °C in pre-warmed HBSS containing calcium and magnesium and then transferred to Attofluor™ imaging chambers. Imaging was performed at 37 °C using a Zeiss Axiovert 200M epifluorescence microscope equipped with a 25× multi-immersion objective (NA 0.8), a CoolLED pE-4000 illumination system, and a PCO Edge 4.2 monochrome camera. jGCaMP8s fluorescence was recorded using 470/20 nm excitation and 525/50 nm emission filters. mRuby2 fluorescence was recorded using 550/15 nm excitation and 580/30 nm emission filters. Images were acquired at 10 Hz for 5 min. Overall fluorescence signals were background-subtracted using mean intensity values from cell-free regions, and individual neurons (soma plus proximal dendrites) were selected as regions of interest (ROIs). jGCaMP8s fluorescence was normalized to mRuby2 fluorescence to correct for motion artifacts and expression levels. Calcium signals were expressed as ΔF/F_0_, where F_0_ represents the mean baseline fluorescence and ΔF the change in fluorescence at each time point relative to F_0_. For pharmacological experiments, spontaneous activity was recorded for 1 min before drug application. Oscillation frequency and amplitude were quantified from ΔF/F_0_ traces obtained between 2 and 4 min after the start of image acquisition. Image processing and data analysis were performed using Fiji/ImageJ.

### 4.10. Viability and Survival Assays

MTT assay: Primary hippocampal neuron cultures were seeded in 24 well plates and analyzed at DIV16. Thiazolyl Blue Tetrazolium Bromide (MTT; Sigma) was added to each well to a final concentration of 0.5 mg/mL. Cultures were incubated for 30 min at 37 °C to allow reduction of MTT to insoluble formazan crystals. Formazan was then solubilized in acidified isopropanol (isopropanol containing 0.04 N HCl), and absorbance was measured at 450 nm using a FluoStar Optima microplate reader (BMG Labtech, Ortenberg, Germany).

LDH assay: Cell membrane damage and cytotoxicity were assessed using a lactate dehydrogenase (LDH) release assay. At DIV16, 2 mM iodonitrotetrazolium chloride, 3.2 mM β-nicotinamide adenine dinucleotide (NAD, sodium salt), 160 mM lithium lactate and 15 μM 1-methoxyphenazine methosulfate (MPMS) in 0.2 M Tris–HCl (pH 8.2) were added to conditioned culture medium (NB or NB+) from hippocampal neurons. After incubating for 30 min at room temperature in the dark, the reaction was stopped by adding 1 M acetic acid, and LDH activity was quantified by measuring absorbance at 490 nm using a FluoStar Optima microplate reader (BMG Labtech). Data from LDH and MTT assays were exported to Excel for analysis and normalized to the corresponding control condition in each experiment.

Live–dead assay: Neuronal viability was assessed using a live–dead assay in primary hippocampal neuron cultures plated in 12-well plates. At DIV16, cultures were incubated in complete Hank’s balanced salt solution containing 1 µg/mL Hoechst 33342 (total nuclei), 1 µM Calcein-AM (viable cells), and 5 µM propidium iodide (PI; dead cells) for 30 min at 37 °C. Cells were then washed with complete Hank’s solution and imaged using a Zeiss AxioObserver7 fluorescence microscope. For each experimental condition, 20 random fields were acquired using a 20× objective, yielding quantitative analysis of approximately 800–1000 cells per condition.

### 4.11. Electrophysiology

Glass coverslips containing hippocampal neuron cultures (DIV15–17) were transferred to an open recording chamber and continuously perfused with artificial cerebrospinal fluid (aCSF) containing (in mM): 119 NaCl, 2.5 KCl, 2.5 CaCl_2_, 1.2 MgCl_2_, 26 NaHCO_3_, 1 NaH_2_PO_4_, and 11 glucose, adjusted to pH 7.4 and 290 ± 5 mOsm. The aCSF was continuously bubbled with carbogen (95% O_2_, 5% CO_2_), and temperature was maintained at 29 °C. Neurons were previously cultured in NB+ medium and infected with Ng-EGFP AAVs. For recordings of AMPA receptor-mediated miniature excitatory postsynaptic currents (mEPSCs), aCSF was supplemented with 100 µM AP5 (NMDA receptor antagonist), 100 µM picrotoxin (GABAA receptor antagonist), and 1 µM TTX to block action potentials. Patch electrodes (4–6 MΩ), pulled from borosilicate glass and containing Ag/AgCl wires, were filled with an internal solution composed of (in mM): 115 CsMeSO_3_, 20 CsCl, 10 HEPES, 2.5 MgCl_2_, 4 Na_2_ATP, 0.4 Na-GTP, 10 sodium phosphocreatine, 0.6 EGTA, and 10 lidocaine N-ethyl bromide, adjusted to pH 7.25 and 290 ± 5 mOsm. Cells were voltage-clamped at −60 mV and recorded for 4–6 min using a gap-free protocol. Signals were acquired with a MultiClamp 700B amplifier, a Digidata 1550B A/D converter, and pCLAMP software version 10 (Molecular Devices, San Jose, CA, USA). mEPSCs were detected and analyzed using Clampfit version 10 (Molecular Devices). For recordings of spontaneous activity and neuronal synchrony, patch electrodes were filled with an internal solution containing (in mM): 115 K-gluconate, 20 KCl, 10 HEPES, 2 MgCl_2_, 4 Na_2_ATP, and 0.3 Na_3_GTP, adjusted to pH 7.2–7.3 and 290 ± 5 mOsm. Spontaneous activity was recorded simultaneously from pairs of neurons in current-clamp mode for 4–6 min using a gap-free protocol. Data were acquired using the same electrophysiological setup and analyzed in MATLAB R2024a. Spike synchrony was quantified following the method described by Quiroga et al. [63], using a coincidence window of ±2.5 ms.

### 4.12. Statistical Analysis

Data were analyzed using GraphPad Prism 8. For parametric comparisons, Student’s *t*-test was used for two groups, and one-way or two-way ANOVA with Bonferroni post hoc correction was used for multiple groups. For non-parametric comparisons, the Mann–Whitney test or Kruskal–Wallis test was applied for two or more groups, respectively. Results are presented as mean ± SEM from at least three independent experiments. Statistical significance is defined as follows: * *p* < 0.05, ** *p* < 0.01, *** *p* < 0.001, **** *p* < 0.0001. The absence of asterisks indicates non-significant differences (ns).

## 5. Conclusions

In summary, our findings indicate that Ng expression during periods of active synaptogenesis promotes morphological differentiation, enhances synaptic connectivity, and facilitates the emergence of synchronized network activity. These effects are accompanied by coordinated adjustments in Ca^2+^/CaM-dependent signaling and synaptic composition that stabilize neuronal output and preserve excitatory balance. At the cellular level, Ng expression promotes metabolic competence and resistance to stress, supporting the development of stable and resilient neuronal networks. Although the present study focuses on developing neuronal cultures and does not directly address Ng function in mature circuits in vivo, the results provide a framework for understanding how CaM-binding proteins coordinate structural development, network activity, and cellular homeostasis. Given that Ng levels decline during aging [21] and in several neurological disorders associated with cognitive impairment, strategies aimed at preserving or restoring Ng expression may help maintain neuronal circuit integrity and mitigate cognitive decline.

## Data Availability

The data sets generated during and/or analyzed during the current study are available from the corresponding author upon reasonable request.

## Supplementary Materials

The following supporting information can be downloaded at https://www.mdpi.com/article/10.3390/ijms27073306/s1.

## Abbreviations

The following abbreviations are used in this manuscript:

2-APB	2-aminoethoxydiphenylborate
AAV	adeno-associated virus
AD	Alzheimer’s disease
AIS	axon initial segment
AnkG	Ankyrin G
AraC	1-beta-arabino-furanosylcytosine
BSA	bovine serum albumin
Ca^2+^	calcium ion
CaM	calmodulin
CaMKII	calcium/calmodulin-dependent protein kinase II
CSF	cerebrospinal fluid
DIV	days in vitro
DMEM	Dulbecco’s modified Eagle’s medium
E19	embryonic day 19
ECL	enhanced chemiluminescence
FBS	fetal bovine serum
HBSS	Hank’s balanced salt solution
LDH	lactate dehydrogenase
LTD	long-term depression
LTP	long-term potentiation
LVs	lentiviral particles
MCI	mild cognitive impairment
mEPSC	miniature excitatory postsynaptic current
MPEP	2-methyl-6-phenylethynylpyridine
MPMS	1-methoxyphenazine methosulfate
MTT	thiazolyl blue tetrazolium bromide
NA	numerical aperture
NAD	β-nicotinamide adenine dinucleotide
NB	neurobasal
Ng	Neurogranin
NMDA	N-methyl-D-Aspartate
PA	phosphatidic acid
PFA	paraformaldehyde
PI	propidium iodide
PKC	protein kinase C
PLL	poly-L-lysine
ROI	region of interest
RT	room temperature
TTX	tetrodotoxin

## References

[1] Represa A., Deloulme J.C., Sensenbrenner M., Ben-Ari Y., Baudier J. (1990). Neurogranin: Immunocytochemical Localization of a Brain-Specific Protein Kinase C Substrate. J. Neurosci..

[2] Alvarez-Bolado G., Rodríguez-Sánchez P., Tejero-Díez P., Fairén A., Díez-Guerra F.J. (1996). Neurogranin in the Development of the Rat Telencephalon. Neuroscience.

[3] Watson J.B., Sutcliffe J.G., Fisher R.S. (1992). Localization of the Protein Kinase C Phosphorylation/Calmodulin-Binding Substrate RC3 in Dendritic Spines of Neostriatal Neurons. Proc. Natl. Acad. Sci. USA.

[4] Gerendasy D.D., Herron S.R., Watson J.B., Sutcliffe J.G. (1994). Mutational and Biophysical Studies Suggest RC3/Neurogranin Regulates Calmodulin Availability. J. Biol. Chem..

[5] Baudier J., Deloulme J.C., Van Dorsselaer A., Black D., Matthes H.W.D. (1991). Purification and Characterization of a Brain-Specific Protein Kinase C Substrate, Neurogranin (P17). Identification of a Consensus Amino Acid Sequence between Neurogranin and Neuromodulin (GAP43) That Corresponds to the Protein Kinase C Phosphorylation Si. J. Biol. Chem..

[6] Randall Slemmon J., Morgan J.I., Fullerton S.M., Danho W., Hilbush B.S., Wengenack T.M. (1996). Camstatins Are Peptide Antagonists of Calmodulin Based upon a Conserved Structural Motif in PEP-19, Neurogranin, and Neuromodulin. J. Biol. Chem..

[7] Slemmon J.R., Feng B., Erhardt J.A. (2000). Small Proteins That Modulate Calmodulin-Dependent Signal Transduction. Mol. Neurobiol..

[8] Huang K.P., Huang F.L., Jäger T., Li J., Reymann K.G., Balschun D. (2004). Neurogranin/RC3 Enhances Long-Term Potentiation and Learning by Promoting Calcium-Mediated Signaling. J. Neurosci..

[9] Hoffman L., Chandrasekar A., Wang X., Putkey J.A., Waxham M.N. (2014). Neurogranin Alters the Structure and Calcium Binding Properties of Calmodulin. J. Biol. Chem..

[10] Zhabotinsky A.M., Camp R.N., Epstein I.R., Lisman J.E. (2006). Role of the Neurogranin Concentrated in Spines in the Induction of Long-Term Potentiation. J. Neurosci..

[11] Kubota Y., Putkey J.A., Waxham M.N. (2007). Neurogranin Controls the Spatiotemporal Pattern of Postsynaptic Ca2+/CaM Signaling. Biophys. J..

[12] Ordyan M., Bartol T., Kennedy M., Rangamani P., Sejnowski T. (2020). Interactions between Calmodulin and Neurogranin Govern the Dynamics of CaMKII as a Leaky Integrator. PLoS Comput. Biol..

[13] Díez-Guerra F.J. (2010). Neurogranin, a Link between Calcium/Calmodulin and Protein Kinase C Signaling in Synaptic Plasticity. IUBMB Life.

[14] Pak J.H., Huang F.L., Li J., Balschun D., Reymann K.G., Chiang C., Westphal H., Huang K.-P. (2000). Involvement of Neurogranin in the Modulation of Calcium/Calmodulin-Dependent Protein Kinase II, Synaptic Plasticity, and Spatial Learning: A Study with Knockout Mice. Proc. Natl. Acad. Sci. USA.

[15] Miyakawa T., Yared E., Pak J.H., Huang F.L., Huang K.P., Crawley J.N. (2001). Neurogranin Null Mutant Mice Display Performance Deficits on Spatial Learning Tasks with Anxiety Related Components. Hippocampus.

[16] Casaletto K.B., Elahi F.M., Bettcher B.M., Neuhaus J., Bendlin B.B., Asthana S., Johnson S.C., Yaffe K., Carlsson C., Blennow K. (2017). Neurogranin, a Synaptic Protein, Is Associated with Memory Independent of Alzheimer Biomarkers. Neurology.

[17] Thorsell A., Bjerke M., Gobom J., Brunhage E., Vanmechelen E., Andreasen N., Hansson O., Minthon L., Zetterberg H., Blennow K. (2010). Neurogranin in Cerebrospinal Fluid as a Marker of Synaptic Degeneration in Alzheimer’s Disease. Brain Res..

[18] Portelius E., Zetterberg H., Skillbäck T., Törnqvist U., Andreasson U., Trojanowski J.Q., Weiner M.W., Shaw L.M., Mattsson N., Blennow K. (2015). Cerebrospinal Fluid Neurogranin: Relation to Cognition and Neurodegeneration in Alzheimer’s Disease. Brain.

[19] Kester M.I., Teunissen C.E., Crimmins D.L., Herries E.M., Ladenson J.H., Scheltens P., van der Flier W.M., Morris J.C., Holtzman D.M., Fagan A.M. (2015). Neurogranin as a Cerebrospinal Fluid Biomarker for Synaptic Loss in Symptomatic Alzheimer Disease. JAMA Neurol..

[20] Kvartsberg H., Duits F.H., Ingelsson M., Andreasen N., Öhrfelt A., Andersson K., Brinkmalm G., Lannfelt L., Minthon L., Hansson O. (2015). Cerebrospinal Fluid Levels of the Synaptic Protein Neurogranin Correlates with Cognitive Decline in Prodromal Alzheimer’s Disease. Alzheimers Dement..

[21] Saunders T., Gunn C., Blennow K., Kvartsberg H., Zetterberg H., Shenkin S.D., Cox S.R., Deary I.J., Smith C., King D. (2023). Neurogranin in Alzheimer’s Disease and Ageing: A Human Post-Mortem Study. Neurobiol. Dis..

[22] Krucker T., Siggins G.R., McNamara R.K., Lindsley K.A., Dao A., Allison D.W., De Lecea L., Lovenberg T.W., Sutcliffe J.G., Gerendasy D.D. (2002). Targeted Disruption of RC3 Reveals a Calmodulin-Based Mechanism for Regulating Metaplasticity in the Hippocampus. J. Neurosci..

[23] Ben-Ari Y. (2001). Developing Networks Play a Similar Melody. Trends Neurosci..

[24] Blankenship A.G., Feller M.B. (2010). Mechanisms Underlying Spontaneous Patterned Activity in Developing Neural Circuits. Nat. Rev. Neurosci..

[25] Lohmann C., Wong R.O.L. (2005). Regulation of Dendritic Growth and Plasticity by Local and Global Calcium Dynamics. Cell Calcium.

[26] Wayman G.A., Impey S., Marks D., Saneyoshi T., Grant W.F., Derkach V., Soderling T.R. (2006). Activity-Dependent Dendritic Arborization Mediated by CaM-Kinase I Activation and Enhanced CREB-Dependent Transcription of Wnt-2. Neuron.

[27] Mons N., Enderlin V., Jaffard R., Higueret P. (2001). Selective Age-Related Changes in the PKC-Sensitive, Calmodulin-Binding Protein, Neurogranin, in the Mouse Brain. J. Neurochem..

[28] Garrido-García A., de Andrés R., Jiménez-Pompa A., Soriano P., Sanz-Fuentes D., Martínez-Blanco E., Díez-Guerra F.J. (2019). Neurogranin Expression Is Regulated by Synaptic Activity and Promotes Synaptogenesis in Cultured Hippocampal Neurons. Mol. Neurobiol..

[29] Siebler M., Köller H., Stichel C.C., Müller H.W., Freund H.-J. (1993). Spontaneous Activity and Recurrent Inhibition in Cultured Hippocampal Networks. Synapse.

[30] Charlesworth P., Cotterill E., Morton A., Grant S.G., Eglen S.J. (2015). Quantitative Differences in Developmental Profiles of Spontaneous Activity in Cortical and Hippocampal Cultures. Neural Develop..

[31] Martzen M.R., Slemmon J.R. (1995). The Dendritic Peptide Neurogranin Can Regulate a Calmodulin-Dependent Target. J. Neurochem..

[32] Bayer K.U., Giese K.P. (2025). A Revised View of the Role of CaMKII in Learning and Memory. Nat. Neurosci..

[33] D’Amelio M., Cavallucci V., Cecconi F. (2010). Neuronal Caspase-3 Signaling: Not Only Cell Death. Cell Death Differ..

[34] Li Z., Jo J., Jia J.-M., Lo S.-C., Whitcomb D.J., Jiao S., Cho K., Sheng M. (2010). Caspase-3 Activation via Mitochondria Is Required for Long-Term Depression and AMPA Receptor Internalization. Cell.

[35] Ertürk A., Wang Y., Sheng M. (2014). Local Pruning of Dendrites and Spines by Caspase-3-Dependent and Proteasome-Limited Mechanisms. J. Neurosci..

[36] Hollville E., Deshmukh M. (2018). Physiological Functions of Non-Apoptotic Caspase Activity in the Nervous System. Semin. Cell Dev. Biol..

[37] Nguyen T.T.M., Gillet G., Popgeorgiev N. (2021). Caspases in the Developing Central Nervous System: Apoptosis and Beyond. Front. Cell Dev. Biol..

[38] Sarić N., Hashimoto-Torii K., Jevtović-Todorović V., Ishibashi N. (2022). Nonapoptotic Caspases in Neural Development and in Anesthesia-Induced Neurotoxicity. Trends Neurosci..

[39] Ledda F., Paratcha G. (2017). Mechanisms Regulating Dendritic Arbor Patterning. Cell. Mol. Life Sci..

[40] Grabrucker A., Vaida B., Bockmann J., Boeckers T.M. (2009). Synaptogenesis of Hippocampal Neurons in Primary Cell Culture. Cell Tissue Res..

[41] Zhong L., Gerges N.Z. (2012). Neurogranin Targets Calmodulin and Lowers the Threshold for the Induction of Long-Term Potentiation. PLoS ONE.

[42] Li L., Lai M., Cole S., Le Novère N., Edelstein S.J. (2020). Neurogranin Stimulates Ca^2+^/Calmodulin-Dependent Kinase II by Suppressing Calcineurin Activity at Specific Calcium Spike Frequencies. PLoS Comput. Biol..

[43] Fernandes D., Carvalho A.L. (2016). Mechanisms of Homeostatic Plasticity in the Excitatory Synapse. J. Neurochem..

[44] Gulyaeva N.V. (2003). Non-Apoptotic Functions of Caspase-3 in Nervous Tissue. Biochem. Mosc..

[45] Li Z., Sheng M. (2012). Caspases in Synaptic Plasticity. Mol. Brain.

[46] Hyman B.T., Yuan J. (2012). Apoptotic and Non-Apoptotic Roles of Caspases in Neuronal Physiology and Pathophysiology. Nat. Rev. Neurosci..

[47] Mukherjee A., Williams D.W. (2017). More Alive than Dead: Non-Apoptotic Roles for Caspases in Neuronal Development, Plasticity and Disease. Cell Death Differ..

[48] Han K.-S., Cooke S.F., Xu W. (2017). Experience-Dependent Equilibration of AMPAR-Mediated Synaptic Transmission during the Critical Period. Cell Rep..

[49] Iñiguez M.A., De Lecea L., Guadano-Ferraz A., Morte B., Gerendasy D., Sutcliffe J.G., Bernal J. (1996). Cell-Specific Effects of Thyroid Hormone on RC3/Neurogranin Expression in Rat Brain. Endocrinology.

[50] Iñiguez M.A., Rodriguez-Peña A., Ibarrola N., Morreale de Escobar G., Bernal J. (1992). Adult Rat Brain Is Sensitive to Thyroid Hormone. Regulation of RC3/Neurogranin mRNA. J. Clin. Investig..

[51] Stefansson H., Ophoff R.A., Steinberg S., Andreassen O.A., Cichon S., Rujescu D., Werge T., Pietiläinen O.P.H., Mors O., Mortensen P.B. (2009). Common Variants Conferring Risk of Schizophrenia. Nature.

[52] Kvartsberg H., Lashley T., Murray C.E., Brinkmalm G., Cullen N.C., Höglund K., Zetterberg H., Blennow K., Portelius E. (2019). The Intact Postsynaptic Protein Neurogranin Is Reduced in Brain Tissue from Patients with Familial and Sporadic Alzheimer’s Disease. Acta Neuropathol..

[53] The European Parliament and the Council of the European Union (2010). Directive 2010/63/EU of the European Parliament and of the Council of 22 September 2010 on the Protection of Animals Used for Scientific Purposes Text with EEA Relevance. Off. J. Eur. Union.

[54] Kaech S., Banker G. (2006). Culturing Hippocampal Neurons. Nat. Protoc..

[55] Gascón S., Paez-Gomez J.A., Díaz-Guerra M., Scheiffele P., Scholl F.G. (2008). Dual-Promoter Lentiviral Vectors for Constitutive and Regulated Gene Expression in Neurons. J. Neurosci. Methods.

[56] McClure C., Cole K.L.H., Wulff P., Klugmann M., Murray A.J. (2011). Production and Titering of Recombinant Adeno-Associated Viral Vectors. J. Vis. Exp. JoVE.

[57] Schneider C.A., Rasband W.S., Eliceiri K.W. (2012). NIH Image to ImageJ: 25 Years of Image Analysis. Nat. Methods.

[58] Schindelin J., Arganda-Carreras I., Frise E., Kaynig V., Longair M., Pietzsch T., Preibisch S., Rueden C., Saalfeld S., Schmid B. (2012). Fiji: An Open-Source Platform for Biological-Image Analysis. Nat. Methods.

[59] Gallego-Garcia C., Martínez Blanco E., Diez-Guerra F.J. (2025). SynapTrack: An Automated Tool for Synapse Quantification.

[60] Grubb M.S., Burrone J. (2010). Activity-Dependent Relocation of the Axon Initial Segment Fine-Tunes Neuronal Excitability. Nature.

[61] Zhang Y., Rózsa M., Liang Y., Bushey D., Wei Z., Zheng J., Reep D., Broussard G.J., Tsang A., Tsegaye G. (2023). Fast and Sensitive GCaMP Calcium Indicators for Imaging Neural Populations. Nature.

[62] Shaner N.C., Campbell R.E., Steinbach P.A., Giepmans B.N.G., Palmer A.E., Tsien R.Y. (2004). Improved Monomeric Red, Orange and Yellow Fluorescent Proteins Derived from *Discosoma* sp. Red Fluorescent Protein. Nat. Biotechnol..

[63] Quian Quiroga R., Kreuz T., Grassberger P. (2002). Event Synchronization: A Simple and Fast Method to Measure Synchronicity and Time Delay Patterns. Phys. Rev. E.

