# Supplementary material for: Neurogranin Promotes Neuronal Maturation and Network Activity Through Ca2+/Calmodulin Signaling
Source: Int J Mol Sci. 2026 Apr 6;27(7):3306. doi: 10.3390/ijms27073306 (PMC13073677; doi:10.3390/ijms27073306)
Supplement: Supplementary file 1 [file ijms-27-03306-s001.zip › ijms-4226356-supplementary.pdf]

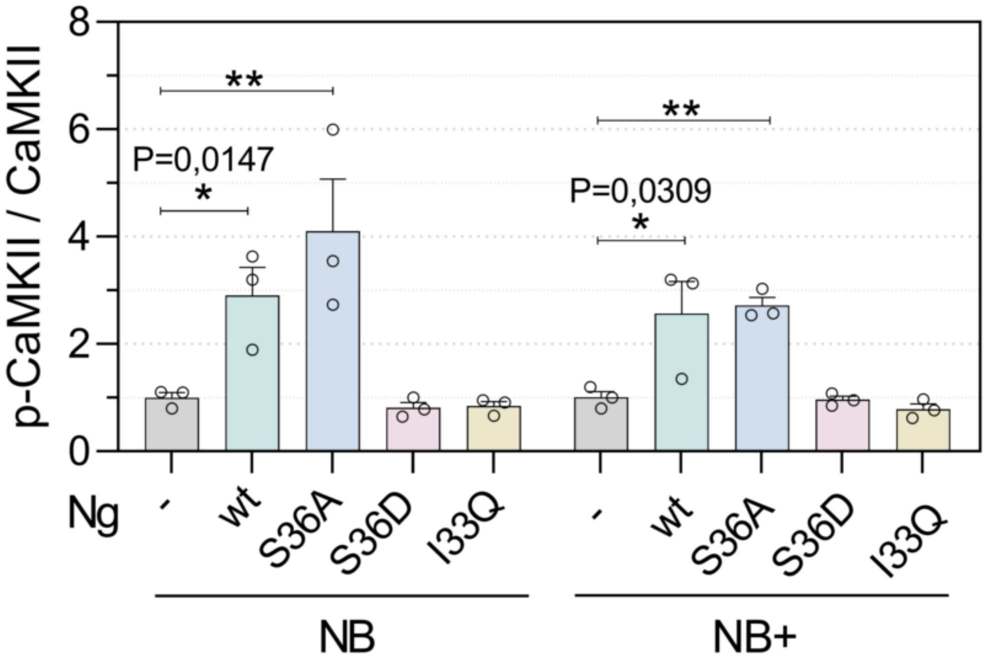

Figure S1: Effect of Ng mutants on the p-CaMKII/CaMKII ratio. Primary hippocampal neurons were infected at DIV7 with AAV-Ng wt or the indicated mutants and maintained in NB or NB+ media. Protein extracts were collected at DIV16 and analyzed by Western blot to quantify phosphorylated CaMKII and total CaMKII. The p-CaMKII/CaMKII ratios were calculated and normalized to non-infected neurons maintained in NB medium. Neurons expressing wild-type Ng or the S36A mutant showed a marked increase in the p-CaMKII/CaMKII ratio, whereas mutants deficient in calmodulin binding did not. (n = 3, mean ± SEM).

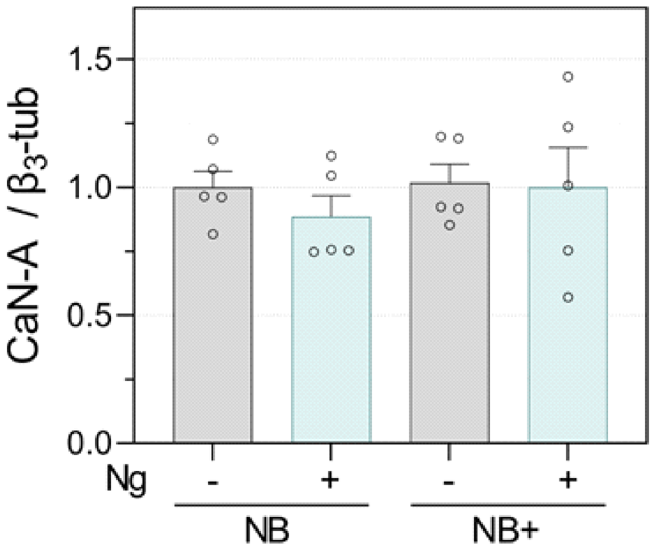

Figure S2: Effect of Ng on Calcineurin-A levels. Cultured hippocampal neurons infected with AAV-Ng at DIV7 and maintained in NB or NB+ medium were extracted at DIV16 and analyzed by Western blot. CaN-A levels were normalized to β3-tubulin. Histogram show mean normalized values relative to non-infected neurons in NB. (n = 5, mean ± SEM).

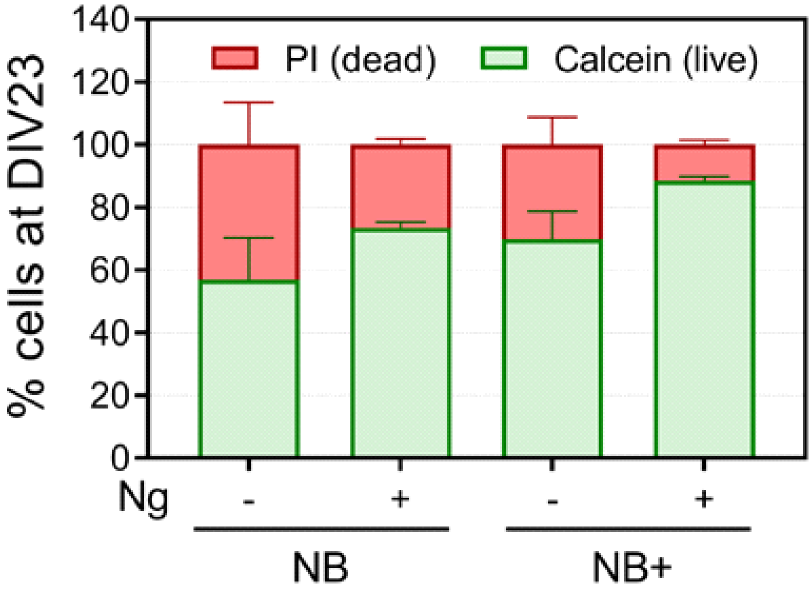

Figure S3: Effect of Ng expression on cell viability in DIV23 cultures. Cell viability was assessed at DIV23 using Calcein-AM, propidium iodide, and Hoechst 33342, as described in Fig. 6c. In Ng-expressing cultures exhibited a higher number of live cells compared with matched control cultures. (n = 3, mean ± SEM).

Plasmids	Backbone	Insert	promoter	expression	tag
**pLV-mRuby2-jGCaMP8s**	pLOX	mRuby & jGCaMP8s	CaMKIIα0.4	Lentiviral	mRuby2
**pAAV-Ng**	**pAAV	Ng	CaMKIIα0.4	AAV2	
**pAAV-Ng-S36A**	pAAV	Ng-S36A	CaMKIIα0.4	AAV2	
**-pAAV-Ng-S36D**	pAAV	Ng-S36D	CaMKIIα0.4	AAV2	
**pAAV-Ng-I33Q**	pAAV	Ng-I33Q	CaMKIIα0.4	AAV2	
**pAAV-Ng-EGFP**	pAAV	Ng	CaMKIIα0.4	AAV2	EGFP
**pFΔ6***		AAV helper		AAV2	
**pRV1***		cap		AAV2	
**pH21***		rep-cap		AAV2	

Table S1: Plasmids developed and used in this study

* Kindly donated by Dr Hilmar Bading (Department of Neurobiology, Interdisciplinary Center for Neurosciences (IZN), Heidelberg, Germany
** pAAV backbones were from Addgene #165429

Plasmids	Addgene	Backbone	Insert	promoter	expression	tag
**pAAV-shNg-mRuby2**	Modified from #92155	pAAV	shRNA targeting Ng	H1	AAV2	mRuby2
**pAAV-shCTRL**	#181875	pAAV	control shRNA	U6	AAV2	mCherry
**pCMVRδ8.74**	#22036		gag pol tat rev		lentiviral	
**pMD2.G**	#12259		VSV-G envelope		lentiviral	

Table S2: Plasmids obtained from Addgene

		Antibody dilution		
**Antibodies**	**Species**	**IF (cell culture)**	**WB**	**Reference**
**Ankiryn-G**	Guinea-pig	1:250		Synaptic System, 386 005
**β_{III}-tubulin**	Mouse		1:50.000	Sigma, T8660
**Bax**	Rabbit		1:2.000	Santa Cruz Biotech., sc-493
**Bcl-2**	Rabbit		1:2.000	Santa Cruz Biotech. sc-492
**CaM**	Mouse		1:5.000	Millipore, 05-173
**CaMKIIα**	Mouse		1:10.000	Millipore, 05-532 6G9
**CaN-A**	Rabbit		1:10.000	Cell Signaling, 2614
**Active caspase-3 (Asp175)**	Rabbit		1:1.000	Cell Signaling, 9961
**eIF2α**	Rabbit		1:2.000	Santa Cruz Biotech. sc-133132
**Phospho-CaMKII (Thr286)**	Rabbit		1:2.000	PhosphoSolutions, p1005-286
**Phospho-eIF2 (Ser52)**	Rabbit		1:2.000	Invitrogen, 44-788G
**GAD65**	Mouse	1:5.000		DSHB Iowa GAD-6
**GAPDH**	Mouse		1:30.000	Millipore, MAB374 6C5
**Gephyrin-Oyster-550**	Mouse	1:500		Synaptic Systems, 147 011 C3
**GluA1**	Rabbit		1:2.000	Millipore, AB1504
**GluA2**	Rabbit		1:2.000	Cell Signaling #13607
**GluN1**	Mouse		1:2.000	Millipore, MAB1586
**GluN2A**	Rabbit		1:2.000	Millipore, AB1555P
**GluN2B**	Mouse		1:2.000	Millipore, MAB52220
**MAP2**	Guinea pig<br>Rabbit<br>Chicken	1:5000<br>1:2.000<br>1:1.000		Synaptic Systems, 188004<br>Millipore AB5622<br>Abcam, ab5392
**mGluR5**	Rabbit		1:500	Sigma-Aldrich, AB5675
**Ng**	Rabbit	1:1000	1:30.000	Millipore, AB5620
**PSD-95**	Mouse	1:1000		Millipore, MAB1596 clone 6G6
**vGluT1**	Guinea-pig	1:15.000		Millipore, AB5905

Table S3: Antibodies used in this study for WB and IF
